# Public Parks in Hong Kong: Characteristics of Physical Activity Areas and Their Users

**DOI:** 10.3390/ijerph13070639

**Published:** 2016-06-28

**Authors:** Bik C. Chow, Thomas L. McKenzie, Cindy H. P. Sit

**Affiliations:** 1 Department of Physical Education, Hong Kong Baptist University, Kowloon Tong, Kowloon, Hong Kong, China; bchow@hkbu.edu.hk; 2 School of Exercise and Nutritional Sciences, San Diego State University, 5127 Walsh Way, San Diego, CA 92115, USA; tmckenzie@sdsu.edu; 3 Department of Sports Science and Physical Education, The Chinese University of Hong Kong, Shatin, New Territories, Hong Kong, China

**Keywords:** built environment, direct observation, exercise, parks, physical activity, recreation, SOPARC, surveillance

## Abstract

Public parks, salient locations for engaging populations in health promoting physical activity, are especially important in high-density cities. We used the System for Observing Physical Activity in Communities (SOPARC) to conduct the first-ever surveillance study of nine public parks in Hong Kong (288 observation sessions during 36 weekdays and 36 weekend days) and observed 28,585 visitors in 262 diverse areas/facilities. Parks were widely used throughout the day on weekdays and weekend days and across summer and autumn; visitor rates were among the highest seen in 24 SOPARC studies. In contrast to other studies where teens and children dominated park use, most visitors (71%) were adults and seniors. More males (61%) than females used the parks, and they dominated areas designed for sports. Over 60% of visitors were observed engaging in moderate-to-vigorous physical activity, a rate higher than other SOPARC studies. Facilities with user fees were less accessible than non-fee areas, but they provided relatively more supervised and organized activities. Assessing parks by age, gender, and physical activity can provide useful information relative to population health. This study not only provides information useful to local administrators for planning and programming park facilities relative to physical activity, but it also provides a baseline for comparison by other high-density cities.

## 1. Introduction

The built environment, including parks, plays a key role in public health [1,2,3,4]. In addition to providing opportunities for people to be outdoors where they can socialize and receive the benefits of fresh air and sunshine, parks provide opportunities for people to participate in health-related physical activity (PA). Engaging in PA regularly is important in the reduction in risk of numerous chronic diseases and to promote mental and psychological wellbeing and is important for people of all ages [5]. The *2008 United States Physical Activity Guidelines* [6], for example, recommend that children and adolescents engage in at least 60 min of mostly moderate-to-vigorous physical activity (MVPA) daily and the World Health Organization (WHO) [7] recommends that adults aged 18–64 participate in at least 150 min of moderate-intensity PA or 75 min of vigorous-intensity PA (or some combination) weekly.

Parks that are accessible to the public regardless of age or income are particularly important in urban areas, especially in high-density cities. Public parks are especially valuable because they offer no- or low-cost programs, are typically open throughout the day, and are designed with recreation facilities that support leisure-time physical activity [8,9]. The frequency of park use and the activities that people do in them vary substantially, and these are associated with numerous factors, including population density, people’s perceptions of safety, and park size, facilities, amenities, programming, and staffing [8,10,11,12,13,14,15].

Parks are of different sizes, and they also have diverse spaces, facilities, and features that may attract people with different characteristics (e.g., gender, age) [11,12,13,14,15]. For example, the National Study of Neighborhood Parks (NSNP), a direct observation investigation of 174 neighborhood parks in 25 randomly selected U.S. cities with a population over 100,000 people found parks disproportionately used by males, children, and teens while being underutilized by adults, especially seniors [11]. Park size matters, and the NSNP study found that relative park size independently contributed to about 10% of the use of these neighborhood parks. Population density and the number of facilities were also strong predictors of park use, with smaller parks tending to have more users per acre [11].

Numerous areas and facilities (e.g., sports fields/courts, exercise stations, walking paths) are designed specifically to engage park users in moderate and vigorous physical activity (MVPA) [16]. Other spaces, such as picnic areas, are more conducive to sedentary activities such as relaxing and socializing. Few evaluations have directly measured specific facility use and documented the degree to which they enhance park use and physical activity [10,13,15,16]. Documenting the association between types of facilities and physical activity in parks can provide information that is useful for building or redesigning parks so that they can best promote population PA. This is particularly important when considering the value of parks to a community relative to the costs of building and maintaining specific facilities [17].

Seasonality is another important consideration in park use and design [10,18]. Not only do weather conditions affect facility use (e.g., outdoor swimming pools), but some sports have traditional “seasons” that attract different numbers and types of participants, including spectators, to specific park areas at different time periods. Finding out how park areas attract various users at different times of the year could potentially influence park design, staffing, and programming in a way that could lead to greater population-level PA. 

While PA can be measured in many different ways, systematic observation excels in being able to provide contextually-rich information on the setting in which activity occurs [19]. Direct observation has the advantages of flexibility, high internal validity, low inference, and low subject burden and is particularly useful for determining how PA is influenced by the immediate environment. During the past decade the System for Observing Physical Activity in Communities (SOPARC) was developed and evaluated [18,20] and is becoming a widely used indicator of park use [10,11]. SOPARC assesses park area users’ PA levels, gender, activity modes/types, and apparent age groupings while simultaneously providing contextual information on the activity area (i.e., accessibility, usability, and levels of supervision and organization). With multiple observations, SOPARC can provide a valid measure of weekly park use and activity levels [11]. Evenson and colleagues [10] recently reviewed 24 cross-sectional studies that used SOPARC and reported that most of the studies were conducted in the USA and during a single season only. The review indicated that the activity levels of park users varied greatly across studies, with youths generally being more physically active than adults and younger children being more active than adolescents. In addition to these cross-sectional studies, SOPARC has been used to assess a variety of interventions including the implementation of community fitness programs [21], modifying policies and programming [17] and adding fitness zones [22] and smaller pocket parks [23]. 

Our review of the literature located only two studies that used SOPARC to assess physical activity and park use in Asian countries, one in Nanching, China [24] and the other in Taipai, Taiwan [25]. Direct observations of park use in Hong Kong, a large metropolitan area that has many residents leading sedentary lifestyles, have not been reported. Hong Kong has approximately 7.3 million residents; and with a land population density of 6690 persons/km^2^ in 2014 is one of the most densely populated cities in the world and has a large proportion of citizens living in high-rise buildings. A recent Behavioral Risk Factor Surveillance System Report [26] indicated that over three-fifths (62.5%) of Hong Kong adults did not reach WHO recommended physical activity levels [7]. 

In addition to having numerous small parks without sports facilities and gardens that cater to more sedentary activities, Hong Kong has 30 urban public parks that are managed by the government Leisure and Cultural Services Department that include at least one outdoor sports facility and one children’s playground. The degree to which these urban parks and their diverse physical activity facilities are used by males and females and by different age groups has not been examined. We used SOPARC methods to objectively quantify the characteristics of the users (e.g., gender, age grouping, physical activity levels) of 262 specific PA areas in nine of the public parks in Hong Kong. To determine potential seasonal influence, we conducted observations in the same parks during both summer (June, July) and autumn (October, November). In addition to providing information useful to Hong Kong administrators for future planning of park facilities and establishing priorities to promote physical activity, the results of the investigation provide a baseline for comparison by other large, high-density cities in Asia and beyond.

## 2. Methods

### 2.1. Park Selection and Target Areas

Table 1 describes the characteristics of the nine selected parks (range: 4–19 hectares) and their surrounding areas in the urban neighborhoods of Hong Kong. We purposely selected a small (4.0–4.5 hectares), medium (7.13–9.79 hectares), and large (14.86–19 hectares) park in the three Hong Kong regions: Hong Kong Island (*n* = 2 parks), Kowloon (*n* = 3 parks), and New Territories (*n* = 4 parks). The number of selected parks was based on the relative population of the three regions according to 2011 Hong Kong census data (i.e., Hong Kong Island: 18.0%; Kowloon: 29.8%, New Territories: 52%). We restricted park selection to avoid those that were in close proximity, and we observed only parks that were (a) within a 15 min walk of a large residential area (>600 flats, see Table 1) and (b) had at least one outdoor sports facility and one children’s playground (see Table 2). The largest park was within a 2 min walk of a mass transit railway, and all others were within an 11–19 min walk from one.

Prior to data collection, parks were mapped and measured and subsequently divided into smaller, specific PA areas that were targeted for observation. Table 2 identifies the number of areas observed by type/facility and the number and proportion of parks with each type. A total of 262 areas were targeted for observation, with the number of areas differing by park size (large = 15–42 areas; medium = 16–27 areas; small = 10–15 areas). Numerous different area types were observed, including some that required a fee for use (e.g., archery range, indoor courts, tennis courts). Each of the nine parks had at least one basketball court, a playground, and a fitness station; only two parks had an indoor sport court and only two had a swimming pool.

### 2.2. SOPARC Instrumentation and Protocol

We assessed the park areas using SOPARC [20], a widely used instrument designed specifically to measure park use and physical activity [10,11] that has been shown to provide a valid measure of park use [18] and to have high inter-observer reliability [4,10,18]. Trained data collectors (*n* = 7) observed all the physical activity areas (total = 262) in the nine parks four times a day during two weekdays and on Saturday and Sunday during both summer and autumn of 2012. Mean temperatures and relative humidity were 30.7 °C and 77.1% during summer and 23.1 °C and 71.1% during autumn. Observations were not made on holidays or during rainy weather; observations postponed due to rain were made up at the same time on the same day of the following week. The four daily observation start times were: early morning (7:00 a.m.), late morning (11:30 a.m.), afternoon (3:00 p.m.), and evening (7:00 p.m.).

Following the procedures of the SOPARC protocol manual [29], during each visit to a target area observers first entered data on the area context (i.e., accessible, usable, supervised, organized) and then scanned systematically from left to right, focusing first on females and then on males (taking approximately 1 s per person). During scanning, observers used a specialized mechanical counter to record the gender, age group (child, age 0–12; teen, age 13–19; adult, age 20–59; senior, age 60 and older), and physical activity level (i.e., sedentary, walking/moderate, vigorous) of each person in the area. The data were then transferred to prepared observation forms. 

### 2.3. Observer Training

Data were collected by seven research assistants trained using the standard SOPARC protocol [29]. They first memorized the operational definitions of the behavioral dimensions and their subcategories and then learned the data recording procedures. Video examples and role-playing were used to demonstrate each category during classroom sessions, and this was followed by both video and field practice observations. Training continued until an observer exceeded an inter-observer agreement score of 80% on video assessment segments. During data collection 32 park visits were observed independently by two data collectors; intraclass reliabilities for people counts ranged from 0.94 to 1.00 with a mean of 0.99, confirming high inter-observer reliability.

### 2.4. Data Analysis

Data were analyzed using SPSS version 23 (IBM SPSS, Chicago, IL, USA) to describe the characteristics of the park areas and their users. Target areas (number, size, type) and specific area characteristics (i.e., accessible, usable, equipped, supervised, organized, and occupied) as well as user characteristics (i.e., gender, age group, physical activity level) were analyzed by size of park (small, medium, large), season (summer, autumn), and the four daily observation periods. 

Chi square statistics were used to determine the differences in frequency counts for female and male park users as well as among the four age categories (child, teen, adult, senior). Observed frequencies for gender and age categories were compared to the 2011 Hong Kong census population data for gender (female, 53.3%; male, 46.7%) and age categories (child, 9.8%; teen, 12.5%; adult, 58.6%; senior, 19.1%) [28]. Physical activity codes were converted to PA intensity levels of metabolic equivalents (METs) based on previous conversion formulae [20,30]. To determine overall MET scores and mean MET values for individuals in park areas we assigned 1.5 METs for sedentary status, 3.0 METs for walking/moderate PA, and 6.0 METs for vigorous PA. 

## 3. Results

### 3.1. Park Visitors

**Figure 1:**
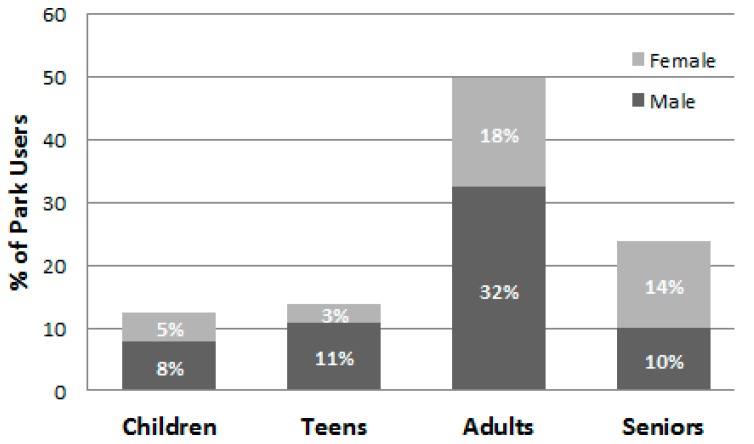
Percent of observed park users by age and gender (*n* = 28,585).

**Figure 2:**
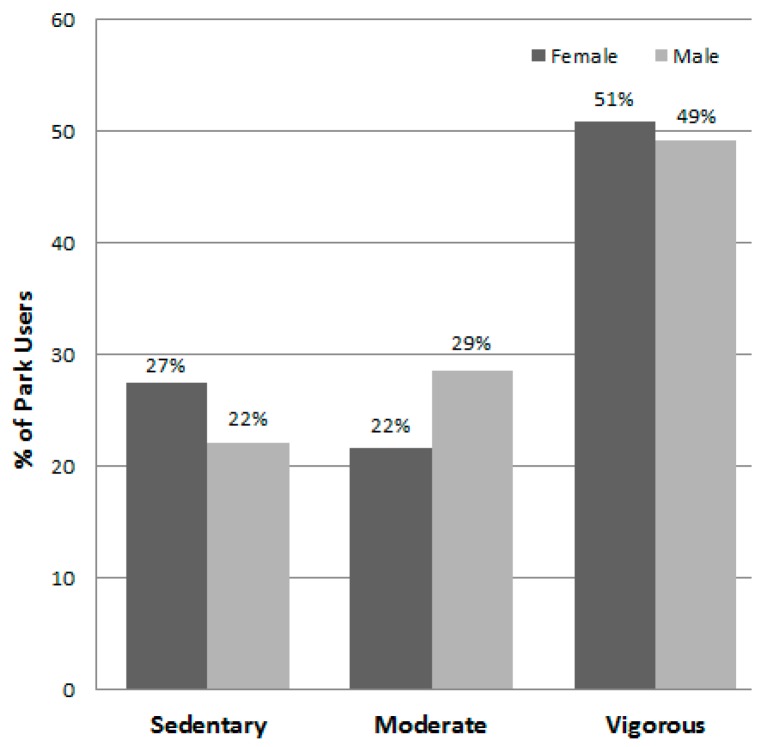
Percent of park users by physical activity levels and gender (*n* = 28,585).

**Figure 3:**
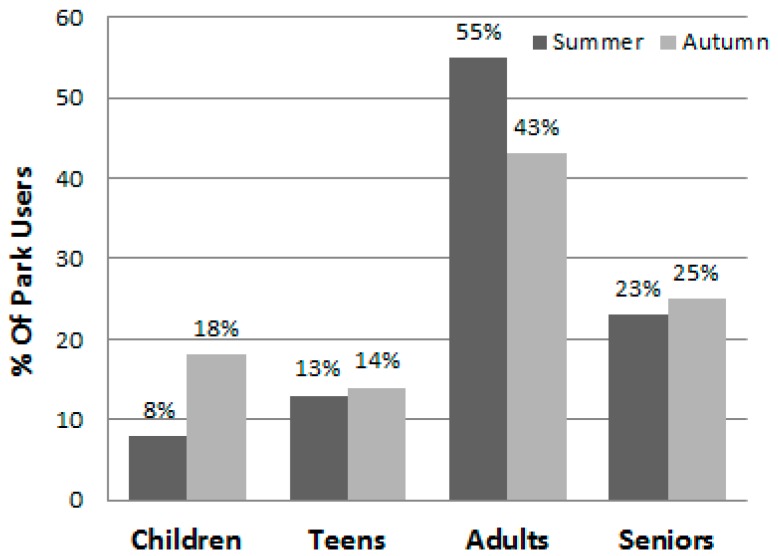
Percent of park users by season and age group (*n* = 28,585).

**Figure 4:**
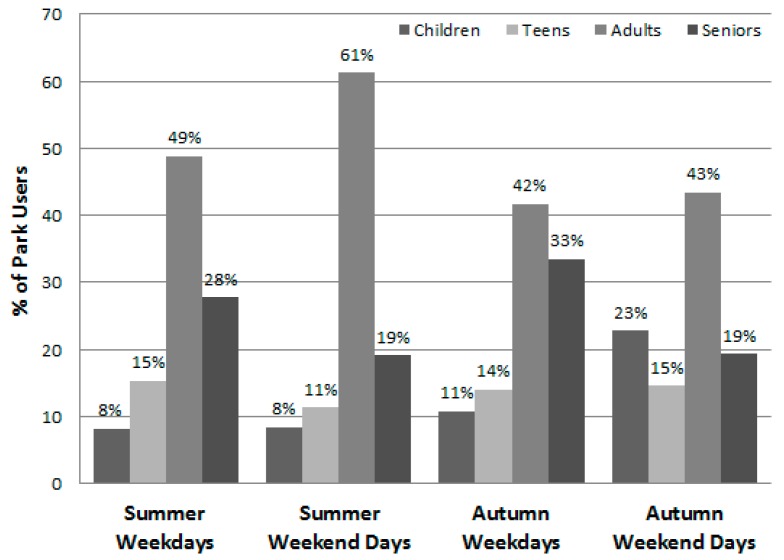
Percent of park users by season on weekdays and weekend days by age group (*n* = 28,585).

Data collectors made a total of 288 visits to the parks (72 days × 4 visits/day), with nine visits (3%) rescheduled due to rain. Table 3 presents the number and proportion of the park users observed by gender, age group, and physical activity level overall and by the individual parks during summer and autumn. Including visitors to the two pools, a total of 28,585 park users were observed and categorized—16,146 (56.5%) during the summer and 12,439 during the autumn (43.5%) (Table 3, Table 4 and Table 5 and Figure 1 and Figure 2). There was substantial variability among the parks in the number of visitors and their characteristics (e.g., the proportion of visitors to a park that were children ranged from 4.0% to 40.7%). Overall, significantly (*p* < 0.001) more males (60.1%) than females were seen in the parks and there were significantly (*p* < 0.001) more adults (45.6%) than seniors (25.6%), children (14.2%), and teens (14.6%). The age group composition of the park users differed by season, with proportionally more children using the parks in autumn and proportionally more adults using them during the summer (Figure 3). Figure 4, which illustrates the proportion of park users by age group during both week days and weekend days, shows that adults were the prominent park users on both weekdays and weekend days and during both seasons.

**Figure 5:**
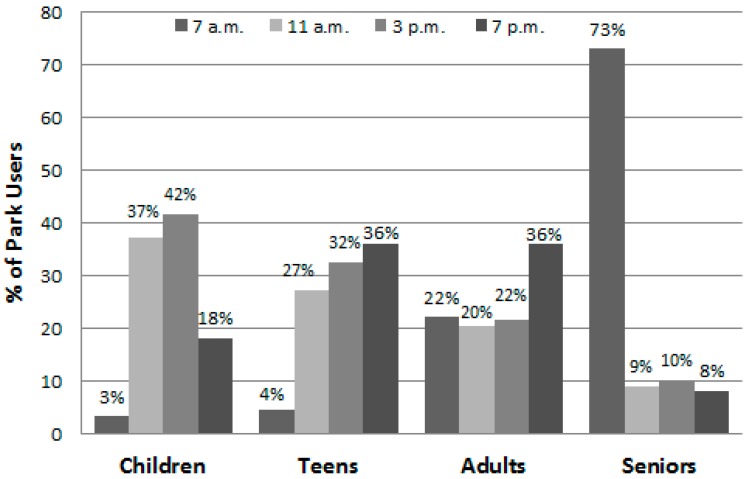
Percent of park users by age group and time of day (*n* = 28,585).

Table 4 summarizes the data for large, medium, and small parks and displays the proportion of observed park users by gender, age group, and physical activity level during weekdays and weekend days. Overall, more people used the larger parks and more visited the parks on a weekend day (57%) than on a weekday (43%) (Figure 4). Park visits were spread throughout the day with 29.4% of park users observed during early morning, 20.7% during late morning, 22.8% during the afternoon, and 27.0% during the evening observation session (Table 4). The fewest people were observed during the 11 a.m and 3 p.m. periods on weekdays (Table 5). Meanwhile, there was substantial variability in the time of day that parks were used by the different age groups (Figure 5). Few children, for example, were in the parks during the 7 a.m. and 11 a.m. observations; meanwhile seniors comprised 73% of those seen during the 7 a.m. session but were about only 9% of those present during the three other observation times.

### 3.2. Park Area Characteristics

**Figure 6:**
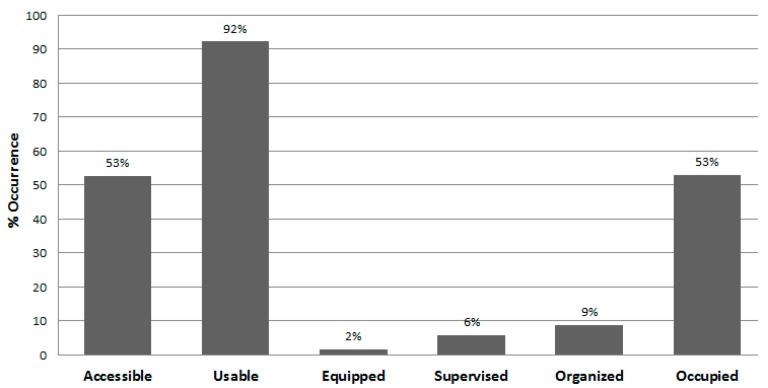
Observed park area characteristics (*n* = 8384 area visits).

**Figure 7:**
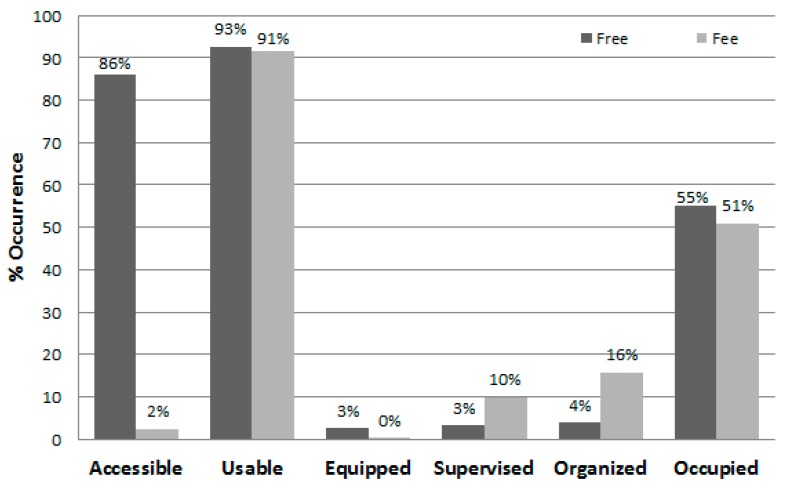
Observed characteristics of free (*n* = 5856 area visits) and fee-charged (*n* = 2528 area visits) park facilities.

Table 6 presents the proportion of observer visits to the free and user-fee areas during which specific contextual characteristics were observed during summer and autumn. Except for the two swimming pools and the archery range that were less available in autumn, the observed characteristics for individual areas were similar during both seasons. In general, facilities that did not require a fee were accessible nearly all the time, while those usually requiring a user-fee were accessible without cost only about 2% of the time. Most facilities were in usable condition nearly all the time during both seasons. Meanwhile, most areas, especially those without an entry fee, were equipped, supervised, or provided organized activities rarely (Figure 6 and Figure 7). Swimming pools, the archery range, and the climbing wall were the areas with the highest levels of supervision. Overall, both free- and fee-based target areas were vacant during about half of the observer visits to the areas, with occupied rates varying substantially by area type. Jogging trails, walking trails, and basketball courts were most frequently occupied free areas and swimming pools (when available) and indoor sport courts were most frequently occupied areas with a user-fee.

Four venues were occupied less than 40% of the time: the archery range, gateball courts, fitness stations, and lawn bowl greens. Children’s playgrounds and fitness stations were in all nine parks, but these venues were frequently empty—46% and 58% of observer visits, respectively. 

### 3.3. Park Areas, Park Users, and Physical Activity

**Figure 8:**
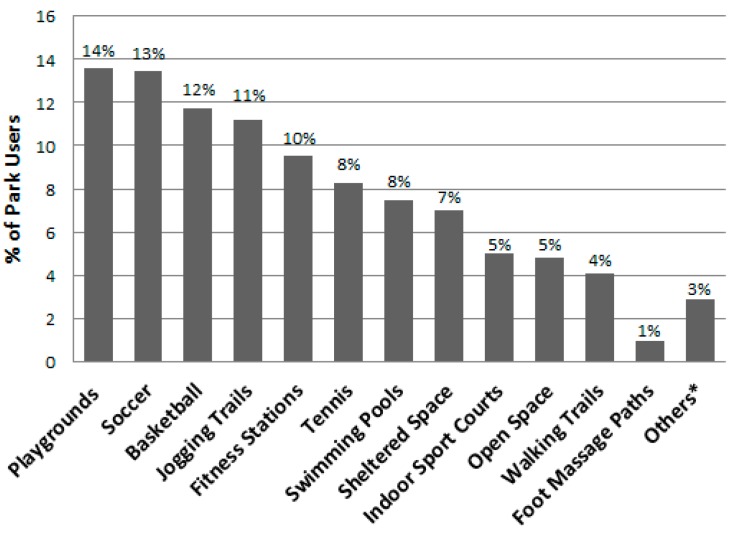
Percent of park area users by area type (*n* = 28,585). * Others include archery range, climbing wall, gateball courts, lawn bowl greens, and roller-skating areas.

**Figure 9:**
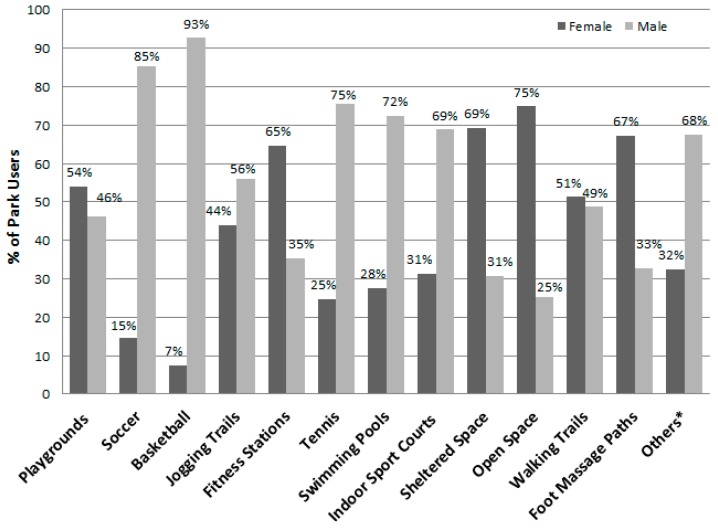
Percent of park area users by gender (total *n*: female = 11,120, male = 17,465). * Others include archery range, climbing wall, gateball courts, lawn bowl greens, and roller-skating areas.

**Figure 10:**
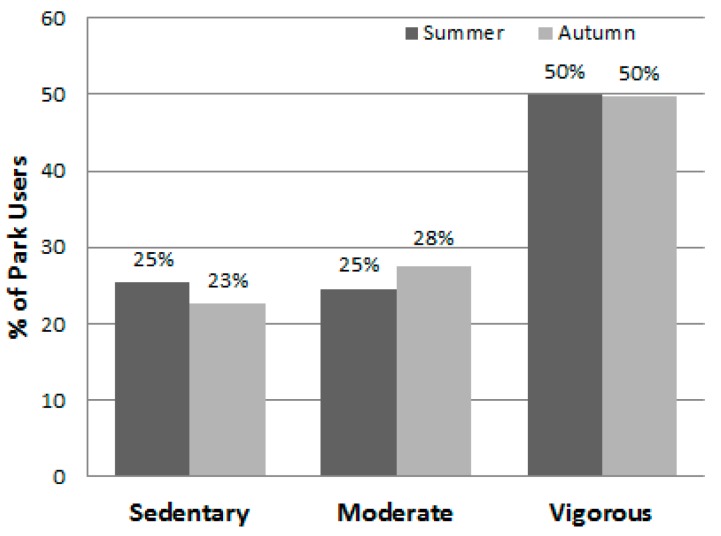
Percent of park users by physical activity levels and season (*n* = 28,585).

Table 7 lists the different park area types in descending order by their total number of users, shows the number of females and males observed, and presents the total METs and MET rate for the area types, overall and by gender. Playgrounds, soccer fields, basketball courts, jogging trails, and fitness stations were the five areas that attracted the most users (see Figure 8) and they had highest estimated energy expenditures (i.e., total METs). Females were observed to engage in significantly less vigorous levels of physical activity (i.e., mean METs per person) than males at playgrounds, on jogging trails, and in swimming pool areas. Figure 9 displays the proportion of individuals using area types by gender and shows that males dominated the use of the sports facilities (i.e., soccer, basketball, tennis, swimming areas, and the indoor sports courts). Meanwhile, proportionally more females used fitness stations, sheltered and open space, and foot massage paths. Overall when observed, 20.7% of the park users were sedentary, 27.0% were walking or in moderate activity, and 52.3% were engaged in vigorous activity (Table 3). Figure 10 shows that the proportions of users engaging in the three different activity levels were similar during the summer and autumn seasons. 

## 4. Discussion

Data for this first-ever surveillance study of nine public parks in Hong Kong were obtained using a validated and widely-used systematic observation instrument during summer and autumn seasons (total of 288 park observation sessions during 72 days, including 36 weekdays and 36 weekend days). A total of 28,585 park visitors in 262 diverse areas, including 2144 swimming pool users, were assessed. The parks were widely used throughout the day on both weekdays and weekend days, with an average of 99 people seen during any single observation period that took less than an hour. The observed mean 367 of visitors per park (range = 131 to 587) during four observation sessions per day is more than the 305 observed in a study of 50 parks in Los Angeles parks [31] and far greater than those in other studies [10,11,32]. Located in density populated areas and within a 15 min walk from at least one large residential area, the visitor rates of these Hong Kong parks were above the 88th percentile of parks in 24 other studies that also used SOPARC [10].

Park size matters. Larger parks, which also had more activity areas, attracted more visitors during the observed periods (small: 3133; medium: 9218; large: 14,090). The exception was Park #3, a large park that had fewer visitors than the medium size parks. Park #3 is located near the center of a relatively large new town in a district with a population density lower than other observed areas. In addition to this large park, the town also has many other smaller green park spaces spread throughout the area. 

A large proportion of the visitors (71%) to the nine parks were adults and seniors, a sharp contrast with the findings of most other studies where teens and children are the typical users [10]. Of special note is the high proportion of visitors who were seniors (25%), especially when compared to the proportion (19.1%) identified in the 2011 Hong Kong population census and to studies that report seniors to be from only 4% [11] to 11% [21] of park users. One exception is an intervention study of green spaces in Taipei, Taiwan that specifically targeted seniors; it reported that 61% of the park users were seniors [25]. It may be that Hong Kong, and possibly other Asian cities, pay specific attention to the welfare of seniors who may have more ambulatory challenges than young people. For example, the Government of Hong Kong Special Administrative Region, which provides guidelines and standards for both passive and active recreation areas, has a specific section governing recreation facilities for the elderly [33]. Eight of the nine parks in the current study had a fitness station area designed specifically for seniors. This explicit attention is important, because seniors, if retired, have increased leisure time and may be attracted to parks if they are designed especially for their needs. Parks that are in close proximity to residential areas and/or accessible to inexpensive mass public transport are also likely to be attractive to seniors. The largest park in the current study was only a 2 min walk from a railway station; it had the most visitors, and 22% of them were seniors. 

Most SOPARC studies conducted outside of Asia report that children and teens are the primary users of neighborhood parks, especially those that have sports facilities [10,11,20]. In the current study, public parks may have been a less attractive venue for young people because Hong Kong youths are often busy with homework and tutorials [34] and may have limited free time to go to parks. As well, schools in Hong Kong often provide on-site organized sport programs and there may not have been a need for students to visit public parks to participate in sports. 

When observed, over 60% of all park users were engaged in MVPA. This is a much higher rate than other studies using the same observation instrument [10,11], including those conducted in two Asian cities [24,25]. Some of this difference can be accounted for the types of PA areas in the parks. We targeted neighborhood parks that included areas specifically designed to accommodate PA, especially sports and physical fitness areas (e.g., basketball and tennis courts, soccer fields, jogging trails). While most other SOPARC studies included these area types, they also included areas that supported more sedentary activities such as picnicking, socializing, and table games [10,11,12,13]. As well, most U.S. parks that have seats or bleachers for spectators, but only two of the Hong Kong target areas had them.

SOPARC studies typically report that more males use parks than females [10,11,18]). Consistent with those findings, more males (61%) were observed in the parks in this study, even though the overall Hong Kong population consists of more females (53.7%) than males (46.7%) (2011 population census). There were substantial gender differences in how most park area types were used, with males dominating the areas designed for competitive sports (e.g., soccer, basketball, tennis).

Overall, the frequency of park use was consistent across the summer and autumn seasons. This seasonal consistency was also found in Los Angeles, where parks were studied year-round. Like Hong Kong, there is not much seasonal temperature variation in Los Angeles. In contrast, locations with substantial temperature changes, also show variations in park use [18,32]. 

The current investigation is one of the first SOPARC studies to assess park areas that were freely accessible and those that required a user fee. Areas with user fees tended to have more supervised (10% vs. 3%) and organized activity (16% vs. 4%) sessions than areas without them. Nonetheless, because the facilities with fees were vastly different (i.e., mainly for competitive sports) than those without, it is unknown how charging a fee may have impacted area use, either overall or by a specific segment of the population (e.g., by low-income families). The fees charged to access these public venues, however, were substantially lower than the fees charged for local private facilities. Meanwhile, the data indicate that both the free- and fee-based areas were frequently used, and given their high usability ratings (about 92%) both were well maintained. 

Several limitations to the study should be noted. The nine parks observed were not a random sample, but they were selected based on park size and on the population census of the three Hong Kong territories. Overall, the selected parks represented approximately 30% of public parks in Hong Kong that had sports facilities. All observer visits to these parks were made during clement weather, so the results represent the best-case scenario for their use. In addition to providing PA, parks and outdoor spaces are important for many other health reasons. We did not explore these reasons or assess why people did or did not use the parks. As well, many parks and green spaces that do not have sport or exercise facilities are scattered throughout Hong Kong, and we did not study these areas or any private sport and exercise facilities. Factors such as these should be considered in the design of future studies, and investigators are encouraged to consider including both intercept interviews in parks and surveys conducted in randomly selected home addresses nearby. 

## 5. Conclusions

The current investigation is the first-ever surveillance study of public parks in Hong Kong. Among its strengths are the use of a validated and internationally-recognized direct observation instrument by trained observers to objectively assess the characteristics of 262 diverse physical activity areas and their use by over 28,000 people during 72 observation days in two seasons. Identifying the differential use of parks and specific park facilities by age, gender, and physical activity levels can provide important information to meet potential needs relative to population health. The study provides information to Hong Kong administrators for planning park facilities and for programming relative to the promotion of PA, especially as it relates to age and gender groupings as well as during the time of day. In addition, the study provides baseline data for comparison by other large, high-density cities in Asia and beyond.

## Figures and Tables

**Table 1:** Characteristics of the nine selected parks and their surrounding areas.

Park ^a^	Size (Hectares) ^b^	Total Target Areas (*n*)	Child Playgrounds (*n*)	Fitness Areas (*n*)	Walk/Jog Trails (*n*)	Sport Courts/Fields (*n*)	Other Areas ^c^ (*n*)	Region ^d^	Nearest Residential Block ^e^		District Size (km^2^) ^f^	District Population Density (*n* per km^2^ ) ^g^
									Type	Total Flats (*n*)		
#1	19.00	45	5	6	1	26	7	HKI	Public	677	10.02	15,477
#2	17.65	51	8	10	1	15	17	Kln	Private	13,213	9.48	40,690
#3	14.86	24	3	7	1	11	2	NT	Private	15,880	138.43	4178
#4	9.79	38	9	8	3	15	3	HKI	Private	12,698	18.90	31,686
#5	8.00	26	1	6	1	16	2	Kln	Public	8800	9.48	40,690
#6	7.13	16	2	1	1	9	3	NT	Public	800	21.82	21,901
#7	4.50	19	3	6	0	8	2	NT	Private	5500	60.70	4918
#8	4.13	24	11	5	1	5	2	NT	Private	6768	136.39	3368
#9	4.00	19	4	6	2	2	5	Kln	Public	5900	9.36	45,181

**^a^** Large parks: #1–#3, Medium parks: #4–#6, Small parks: #7–#9; **^b^** Leisure Cultural & Services Department [27]; **^c^** Other target areas include open space, sheltered space, foot massage paths, swimming pools, and climbing wall; **^d^** Hong Kong has three geographical regions: HKI = Hong Kong Island, Kln = Kowloon, NT = New Territories; **^e^** Nearest residential building block to the park (all within a 15 min walk); **^f^** Hong Kong has 18 total districts, geographically divided as political areas; **^g^** District population density based on 2011 census [28].

**Table 2:** Target areas observed (total *n* = 262) and number (proportion) of parks containing each type.

Target Areas	Description	Parks (*n* = 9) with Area Type	Area Type Observed
		*n* (%), Specific Park	*n* (Range within Parks)
Archery Range	Outdoor area designed for archery (fee)	1 (11%), Park: #9	1 (0–1)
Basketball Court	Outdoor court designed for basketball	9 (100%), Park: All	24 (1–5)
Climbing Wall	Outdoor wall designed climbing (fee)	1 (11%), Park: #5	1 (0–1)
Fitness Station	Outdoor area with fixed fitness equipment	9 (100%), Park: All	55 (1–10)
Foot Massage Path	Outdoor path with cobbled stones for foot massage	5 (56%), Park: #1, #6–#9	8 (1–3)
Gateball Court	Outdoor greenery area designed for gateball	4 (44%), Park: #2, #3, #7, #8	5 (1–2)
Indoor Sport Court	Indoor court, with permanent markings (e.g., for badminton, basketball, handball, volleyball) (fee)	2 (22%), Park: #2, #5	5 (2–3)
Jogging Trail	Outdoor trail designed for jogging	6 (67%), Park: #1, #2, #4, #5, #8, #9	9 (1–3)
Lawn Bowl Green	Outdoor turf area designed for lawn bowling (fee)	1 (11%), Park: #1	2 (0-2)
Open Space	Outdoor spacious area, not-sheltered or marked, but available for exercise and physical activity	7 (78%), Park: #1–#6, #8	11 (1–3)
Playground	Outdoor area with fixed equipment in block (e.g., slides, climbing apparatus) and/or single pieces (e.g., swings) designated for children	9 (100%), Park: All	46 (1–11)
Roller Skating Rink	Outdoor area designed for skating/rollerblading	2 (22%), Park: #2, #8	3 (1–2)
Sheltered Space	Covered non-designated or marked area, useful for exercise and physical activity	5 (56%), Park: #1, #2, #4, #5, #9	18 (1–12)
Soccer Field-Hard Court	Outdoor hard court area, equipped with soccer goals	6 (67%), Park: #1–#3, #5, #6, #8	12 (1–6)
Soccer Field-Turf	Outdoor area of natural grass or artificial turf, equipped with soccer goals (fee)	3 (33%), Park: #4, #5, #7	4 (1–2)
Swimming Pool	Outdoor pool (fee)	2 (22%), Park: #1, #2	6 (2–4)
Tennis Court	Outdoor court, equipped for tennis (fee)	7 (78%), Park: #1–#7	50 (4–14)
Walking Trail	Paved/concrete path designed for walking	2 (22%), Park: #3, #6	2 (0–1)

**Table 3:** Number and proportion of observed park users by gender, age group, and physical activity level overall and in each park (total visitors = 28,585).

Park (Total Visitors)	Gender		Age Group				Activity Level		
	Female (%)	Male (%)	Child (%)	Teen (%)	Adult (%)	Senior (%)	Sedentary (%)	Walking (%)	Vigorous (%)
#1-Large (7947)	39.8	60.2	9.8	8.1	60.1	22.0	36.0	19.3	44.7
#2-Large (6157)	41.5	58.5	8.4	14.4	50.4	26.8	24.7	25.2	50.1
#3-Large (2130)	36.0	64.0	15.4	27.1	32.3	25.2	10.4	33.7	55.9
#4-Medium (3805)	38.0	62.0	19.6	11.9	43.7	24.8	20.9	20.9	58.2
#5-Medium (2974)	28.1	71.9	4.0	27.5	60.0	8.5	14.0	31.4	54.6
#6-Medium (2439)	42.9	51.1	10.9	8.7	51.6	28.8	19.2	48.7	32.1
#7-Small (923)	33.4	66.6	12.0	15.7	37.4	34.9	33.5	19.8	46.7
#8-Small (1707)	43.9	56.1	40.7	7.7	21.9	29.7	16.3	22.5	61.2
#9-Small (503)	49.9	50.1	6.8	10.1	53.3	29.8	11.3	21.5	67.2
Overall Mean	39.3	60.1	14.2	14.6	45.6	25.6	20.7	27.0	52.3

**Table 4:** Proportion of park users by gender, age group, and physical activity levels for large (*n* = 3), medium (*n* = 3), and small (*n* = 3) parks during four daily time periods on 36 weekdays (WD) and 36 weekend days (WE).

Park	Female (%)	Male (%)	Child (%)	Teen (%)	Adult (%)	Senior (%)	Sedentary (%)	Walking (%)	Vigorous (%)
	WD, WE	WD, WE	WD, WE	WD, WE	WD, WE	WD, WE	WD, WE	WD, WE	WD, WE
**All Parks**									
7 a.m.	57.9, 54.4	42.1, 45.6	1.4, 1.4	2.5, 1.5	33.9, 41.6	62.2, 55.5	16.0, 21.1	21.0, 23.2	63.0, 55.7
11 a.m.	35.7, 32.1	64.3, 67.9	25.5, 21.3	21.1, 16.5	38.7, 54.2	14.7, 8.0	28.2, 24.5	29.1, 28.8	42.7, 46.7
3 p.m.	35.2, 36.3	64.8, 63.7	15.6, 26.1	28.3, 15.6	41.3, 49.5	14.8, 8.8	21.7, 31.7	30.8, 22.1	47.5, 46.2
7 p.m.	27.6, 26.0	72.4, 74.0	7.1, 9.7	19.0, 17.3	65.7, 67.2	8.2, 5.8	28.1, 23.0	27.1, 30.5	44.8, 46.5
**Large**									
7 a.m.	56.9, 55.0	43.1, 45.0	1.6, 1.3	0.7, 1.8	36.3, 45.5	61.4, 51.4	18.2, 26.7	17.8, 24.0	64.0, 49.3
11 a.m.	36.8, 33.4	63.2, 66.6	16.5,16.4	20.9, 14.2	44.6, 62.5	18.0, 6.9	30.8, 30.3	24.5, 28.9	44.7, 40.8
3 p.m.	31.8, 39.9	68.2, 60.1	13.8, 20.6	22.2, 14.1	46.6, 54.7	17.4, 10.6	26.6, 37.2	28.5, 21.0	44.9, 41.8
7 p.m.	28.0, 26.9	72.0, 73.1	3.9, 11.7	19.9, 22.2	67.4, 60.5	8.8, 5.6	31.7, 25.7	21.1, 26.8	47.2, 47.5
**Medium**									
7 a.m.	59.2, 51.9	40.8, 48.1	1.2, 2.3	6.9,1.4	33.1, 39.3	58.8, 57.0	11.1, 11.2	27.5, 25.1	61.4, 63.7
11 a.m.	33.3, 28.3	66.7, 71.7	18.9, 19.3	29.0, 22.3	41.1, 48.4	11.0, 9.6	27.4, 15.8	33.3, 32.1	39.3, 52.1
3 p.m.	41.0, 29.4	59.0, 70.6	12.3, 26.1	39.9, 18.6	36.4, 49.3	11.4, 6.0	15.0, 23.6	37.6, 26.9	47.4, 49.5
7 p.m.	26.6, 24.7	73.4, 75.3	12.3, 6.6	15.6, 9.4	65.8, 78.4	6.3, 5.6	23.5, 19.2	35.7, 36.9	40.8, 43.9
**Small**									
7 a.m.	60.1, 56.3	39.9, 43.7	0.4, 0.2	0.6, 0.4	23.7, 28.7	75.3, 70.7	17.7, 15.7	20.9, 15.9	61.4, 68.4
11 a.m.	37.1, 36.0	62.9, 64.0	70.1, 47.1	5.2, 11.5	13.4, 33.3	11.4, 8.1	21.3, 22.5	35.7, 20.0	43.0, 57.5
3 p.m.	32.0, 35.8	68.0, 64.2	43.6, 55.4	16.7, 15.7	27.6, 21.5	12.1, 7.4	18.6, 24.9	15.4, 14.5	66.0, 60.6
7 p.m.	29.3, 24.9	70.7, 75.1	6.3, 10.0	29.0, 19.3	52.0, 62.7	12.7, 8.0	23.7, 20.9	30.3, 27.6	46.0, 51.5
**Overall Mean**	39.3, 36.9	60.7, 63.1	16.7, 18.1	17.2, 12.6	40.7, 48.8	25.4, 20.6	22.1, 22.8	27.4, 25.0	50.5, 52.2

**Notes**: Observed park visitor comparisons; Days of the week: 36 weekdays, *n* = 12,377 (43.0%); 36 weekend days, *n* = 16,208 (57.0%); Daily time periods: 7 a.m., *n* = 8411 (29.4%); 11 a.m., *n* = 5916 (20.7%); 3 p.m., *n* = 6526 (22.8%); 7 p.m., *n* = 7732 (27.0%); Park size: Large, *n* = 6967 (WD) + 9267 (WE) = 16,234; Medium, *n* = 4127 (WD) + 5091 (WE) = 9218; Small, *n* = 1283 (WD) + 1850 (WE) = 3133.

**Table 5:** Number of park visitors during four daily time periods on weekdays and weekend days.

Days	7 a.m.	11 a.m.	3 p.m.	7 p.m.
Weekdays (*n* = 36)	4505	1908	1972	3992
Weekend days (*n* = 36)	3906	4008	4554	3740
TOTAL	8411	5916	6526	7732

**Table 6:** Proportion of observations in park areas during which specific characteristics were observed during summer (SU) and autumn (AU) (total area visits = 8384).

Park Area	Accessible * %		Usable %		Equipped %		Supervised %		Organized %		Occupied %	
	SU	AU	SU	AU	SU	AU	SU	AU	SU	AU	SU	AU
**Free Use Areas (*n* = 5856 visits)**												
Playgrounds	93	92	94	92	7	1	4	1	4	3	48	58
Soccer (hard courts)	85	88	91	87	8	8	9	8	26	12	65	58
Basketball	93	90	99	96	0	0	0	1	11	2	80	64
Jogging Trails	98	95	98	95	0	0	0	0	0	0	87	84
Fitness Stations	97	100	98	100	5	0	0	0	1	0	42	42
Sheltered Space	98	95	99	98	0	6	0	6	6	7	64	49
Open Space	78	84	81	84	1	1	1	0	6	1	48	53
Walking Trails	100	94	100	90	0	0	0	0	0	0	84	65
Foot Massage Paths	99	100	99	100	13	0	0	0	0	0	41	43
Gateball Courts	21	14	95	51	1	0	0	3	0	3	16	24
Roller Skating Rinks	90	92	98	92	4	0	4	31	0	2	42	43
Overall: Free Use Areas	86	86	96	90	4	2	2	5	5	3	56	53
**User-Fee Areas (*n* = 2528 visits)**												
Archery *	0	0	100	100	0	0	25	0	6	0	19	25
Climbing Wall *	0	0	100	100	0	0	25	0	13	25	37	50
Indoor Sport Courts *	6	1	99	96	0	1	0	3	38	39	72	80
Lawn Bowl *	0	0	100	97	0	0	0	0	16	6	34	44
Soccer (turf fields) *	14	0	100	53	0	0	0	3	17	20	52	30
Swimming Pool *^,~^	0	0	88	75	2	0	63	19	6	0	83	75
Tennis *	4	9	94	78	0	0	0	0	19	17	60	49
Overall: User-Fee Areas *	3	1	97	86	0	0	16	4	16	15	51	50

* Area typically required a fee for use; ^~^ Swimming pools were unoccupied in Park #3 when the pools were closed at 11 a.m. Pools in Park #1, were closed during autumn season due to re-construction.

**Table 7:** Number of observed area visitors, PA intensity, and PA intensity by person and by gender.

Park Area	All			Females			Males			*p*-Value (Sig.)
	*n*	Total METs	Mean METs per Person	*n*	Total METs	Mean METs per Female	*n*	Total METs	Mean METs per Male	
Playgrounds	3877	16,814	4.3	2091	8816	4.2 **^a^**	1786	7998	4.5 **^a^**	0.002
Soccer	3841	14,880	3.9	562	2590	4.6	3279	12,290	3.8	
Basketball	3357	14,364	4.3	247	1155	4.7	3110	13,209	4.3	
Jogging Trails	3203	14,384	4.5	1407	6140	4.4 **^a^**	1796	8244	4.6 **^a^**	0.008
Fitness Stations	2717	11,483	4.2	1758	6914	3.9	959	4569	4.8	
Tennis	2367	9834	4.2	582	2562	4.4	1785	7272	4.1	
Swimming Pools	2144	8916	4.2	592	2342	4.0 **^a^**	1552	6575	4.2 **^a^**	0.001
Sheltered Space	1921	7704	4.0	1331	5337	4.0	590	2367	4.0	
Indoor Courts	1531	6374	4.2	478	2024	4.2	1053	4350	4.1	
Open Space	1375	6327	4.6	1029	4778	4.6	346	1550	4.5	
Walking Trails	1164	36,615	3.2	597	1862	3.1	567	1800	3.2	
Foot Massage Path	266	880	3.3	179	595	3.2	87	285	3.3	
Others **^b^**	822	2540	3.1	267	782	2.9	555	1758	3.2	

**^a^** Mean METs per person observed for females was significantly smaller than mean METs per person observed for males at the 0.05 level of significance. **^b^** Other areas included one archery range, one climbing wall, five gateball courts, two lawn bowl greens, and three roller skating rinks with total observed sessions for each area being 32, 32, 128, 32, and 64, respectively.
